# Self-Reported Physical Activity, Injury, and Illness in Canadian Adolescent Ski Racers

**DOI:** 10.3389/fspor.2020.00032

**Published:** 2020-04-28

**Authors:** Patricia K. Doyle-Baker, Carolyn A. Emery

**Affiliations:** ^1^Faculty of Kinesiology, Sport Injury Prevention Research Centre, University of Calgary, Calgary, AB, Canada; ^2^School of Architecture, Planning and Landscape, University of Calgary, Calgary, AB, Canada; ^3^The Alberta Children's Hospital Research Institute, University of Calgary, Calgary, AB, Canada; ^4^Departments of Pediatrics and Community Health Sciences, Cumming School of Medicine, University of Calgary, Calgary, AB, Canada

**Keywords:** adolescents, ski racers, physical activity participation, injury, hospital visits

## Abstract

Youth ski racers spend a considerable amount of time on snow and this may detract from other activities known to influence fundamental movement skills and overall health related outcomes. Parents of racers (*n* = 52 F; *n* = 44 M; age range 9–14 years) registered in the Canadian club system completed a baseline medical questionnaire during preseason testing in 2017. We describe physical activity volume and sport participation outside of physical education classes over the previous 12 months and report on injuries, medication use and health care utilization. The mean number of activities participated was five (range 1–14) with cycling, hiking, and swimming as the preferred choice and a cumulative mean of just under 400 h of activity was reported (range F 27–1,015; M 62–869 h/year) in the past year. During the past 12 months 16% of the athletes reported being injured and injury severity impacted return to sport with range of reported days missed from 1 to 365 days. Thirteen non-concussive injuries were reported in alpine skiing and females (12%, 6/52) reported more lower limb injuries than males (7%, 3/44). More males were concussed over their lifetime, with alpine skiing accounting for 46% and mountain biking 15%. Most athletes (85%) did not take medication on a regular basis and those that did had a medical diagnosis. The frequency of respiratory conditions was 13% (12/96) with males reporting slightly more cases than females. No difference in emergency visits occurred (25%) between males and females in the past 12 months, however females reported more (*n* = 102) allied health care, sport medicine and x-rays appointments when compared to males (*n* = 65). In summary, a high volume of physical activity (an hour plus per day) over the previous 12 months was reported with racers participating in several activities outside of skiing, likely honing their fundamental movement skills. Close proximity to the mountains may have influenced their choices of activity outside of ski racing, and their injuries and a variety of health conditions were typical of their age group. Future research employing wearable technology to objectively quantify the volume and intensity of physical activity participation is recommended.

## Introduction

Alpine ski racers as young as 10–14 years spend a considerable amount of time on snow during the ski season. This may detract from other activities known to influence fundamental movement skills (FMS) and overall health related outcomes. The Canadian Alpine Long-term Athlete Development model (LTAD) was recently redesigned in 2019 with the emphasis toward a more staged development so as to help younger ages achieve both athlete success and continued life-long participation in the sport (https://ltad.alpinecanada.org). The Training and Competition Volume Matrix within the LTAD identifies volumes and quantities associated with performance programs, however it does not address the volume of physical activity (PA) outside the ski training paradigm. Inclusion of all PA and other sports young ski racers participate in should be considered since this added exposure may contribute positively or negatively to their health and performance.

A review of the literature supports that FMS are associated with PA participation (Williams et al., 2008), however, failure to develop these skills during childhood may impact a child's future physical activity level in adulthood (Lloyd et al., 2014; Henrique et al., 2016; Jaakkola et al., 2016). While these studies focus on the relationship between FMS and PA participation, only a few others have gone beyond and identified health indicators (Tremblay et al., 2016; Canadian Physical Activity Guidelines, 2018). Yet, an underlying belief exists that most health indicator benefits occur through PA and sport participation in youth.

Typical health indicators, such as waist circumference, body weight and body mass index (BMI), have been associated positively with FMS (Lubans et al., 2010; Robinson et al., 2015; Duncan et al., 2016; O'Brien et al., 2016) and with sport participation although the direction of the relationship is not clear (Cairney and Veldhuizen, 2017; Comeau et al., 2017). Ski racers for example tend to be heavier than age matched non-athletes, as larger anthropometric characteristics are advantageous in alpine ski racing (Raschner et al., 1995). However, in terms of both physical and mental health indicators other than those related to body composition, not much information is available in the 10–14 year-old ski racing group.

Recently Müller et al. (2017a,b), published a study that examined the incidence, prevalence, and severity of traumatic and overuse injuries and illnesses of elite youth ski racers, 15 years and under. The authors reported a relatively low injury incidence (traumatic, IR = 0.86/1,000 h of training; overuse, IR = 0.28/1,000 h) and high annual illness prevalence (2.4/athlete) in youth ski racers. In addition to this, the knee was the most commonly affected body part (traumatic knee injuries 36.5%), a high annual prevalence of overuse injuries (82%) and prevalence of bone fractures was high (46%) and 66% of the illnesses reported were respiratory tract infections. Overuse injuries and respiratory diseases, although not traditionally thought of as health indicators certainly influence an adolescent's health.

Reliable data collection through injury and illness surveillance methods in youth alpine ski racers remains a challenge (Spörri et al., 2017). In Canada, the independent operation of ski clubs leads to significant difficulties for injury and illness surveillance. The Public Health Agency of Canada reported that alpine skiing is the third-leading cause after hockey and snowboarding for emergency departments visit in children. Furthermore, skiing (12.6%) had the highest rate of hospitalization when compared to snowboarding (11.3%) and hockey (3.6%) (Warda and Yanchar, 2012).

Head injuries, although thought to be less common are noteworthy, since a child's brain is still developing and a brain injury such as a concussion, impacts them with a range of negative consequences, including headaches, depression, memory problems and poor school performance. Increased concussion recognition has many athletes seeking a diagnosis and as such emergency department visits for sport-related concussions doubled in children from 1997 to 2007 (Bakhos et al., 2010). In a recent study of elite youth ski racers, 28.8% of the injuries reported (19/66) were related to the head and 84% (16/19) of these were concussions during the 2016/2017 ski season (Anderson et al., 2019).

Recently, developmental disorders with similar symptomology as listed above for concussions have been associated with increased injury proneness (Nelson et al., 2016). Given that concussions are emerging as an issue in alpine skiing, where cumulative impacts to the head is not uncommon, more research is needed to rule out the possibility of the interactive effects of this combination. According to Pol et al. (2019) a better understanding of how sport injuries occur is warranted with consideration toward adopting a larger health focus so as to improve prevention for medical, economic, scientific and sports success reasons.

The primary aim of this study was to describe the participation of all sport and physical activity by type and volume, and the secondary aim was to provide insight into the injuries reported and health indicators (health care utilization, illness, medication use) in adolescent youth ski club racers in Western Canada.

## Methods

### Participants and Design

Alberta Alpine Ski Association (AASA) is the provincial governing body for alpine ski-racing, and guides standards for programs for over 4,800 athletes, coaches, officials, and volunteers throughout the province of Alberta in Canada (http://albertaalpine.ca/). Within the AASA are numerous ski-clubs, that are privately managed through parent volunteers and/or respective coach hires. Athletes from 5 ski clubs registered in the AASA in the under 12 and 14 (U12, U14) age group categories participated in this study. Participating skiers had been previously recruited for a pre-experimental study during the 2017 pre-season dryland training period on balance agility, and strength exercises (BASE) that required a sample size of *n* = 93 to achieve 80% power at an alpha level of 0.05 (Doyle-Baker et al., 2017). A baseline medical questionnaire was included in the package with other study forms and sent home to the parents prior to dryland fitness testing. The self-report questionnaire included a mixture of question types (single and multiple response, rating scales, true/false statements and open-ended) and required 10–15 min to complete and most parents did so at home. A total of 96 questionnaires were returned (*n* = 52 F; *n* = 44 M), with 4 forms that had incomplete sections.

### Ethics Statement

Athletes and parents were informed of the study aims, requirements, and risks before providing written and verbal participant assent, and written parental consent. The study was performed according to the Declaration of Helsinki. The research was approved by the Conjoint Health Research Ethics Board of the University of Calgary (REB:16-1818).

### Questionnaire Details

The baseline medical questionnaire was developed from an injury surveillance system adapted for high school basketball from the Canadian Intercollegiate Sports Injury Registry (CISIR) (Emery et al., 2007). The questionnaire has been previously used in other studies as a baseline for sport and recreation participation and injury at the University of Calgary in Alberta (Richmond et al., 2016). In this current study we were interested in identifying all other activities ski racers participated in, past injury and injury types from all activities, past diagnosis related to various conditions (bone fracture, systemic diseases, respiratory, circulation or heart, neurological disorder, headaches), medication and supplement use, and health care utilization (practitioner and hospital visits).

The questionnaire asked for detailed information on frequency, hours, and number of weeks of participation across 38 activities, not including physical education (PE) class, over three time periods. The first time period was related to the 6 weeks prior to the start of the preseason dryland training program, the second was related to the past year, i.e., previous 12 months, which matches the time frame from the Canadian General Social Survey, and the third didn't specify a time duration and was considered to be over a lifetime. Within the questionnaire, injury history was based on date, activity, type, body part, treatment and return to sport (RTS) and was divided into concussion (defined as either diagnosed or not or been “knocked out” or had their “bell rung”), non-concussive injury (defined as requiring medical attention or at least 1 day of missed participation in the past 12 months), and any injury not completely healed.

The data was de-identified and entered into REDCap, a secure web-based application by research assistants and the PI cleaned and checked the data (Harris et al., 2009). Where necessary the paper copy of the questionnaire was reviewed, and/or phone call follow up check with the parents occurred.

### Statistical Analysis

Appropriate measures of central tendency are reported [means and 99% confidence intervals (99% CI)] for participant characteristics from the BASE study by sex: age, height, weight, WC, BMI and Predicted VO_2max_. A crude analysis was carried out to assess the distribution of raw data using the Kolmogorov-Smirnov test, which demonstrated all data to have a normal distribution (*p* > 0.05). The majority of the PA and health variables are described as rates, means, [standard deviations (±)] or frequencies (%). Data was analyzed using IBM SPSS 24 software and STATA V.15.

## Results

Ski racing uses the 1st of January as the birth month for the yearly cut-off date for grouping the various competition categories. This relative age effect (RAE) is present in all age categories at both national, as well as at international levels in skiing (Müller et al., 2015). However, in this study the chronological age was calculated based on the cut-off date for collecting the questionnaires.

### Participant Characteristics

A total of 96 racers (M = 44; F = 52) born between 2004 and 2008 (2004 *n* = 20; 2005 *n* = 20; 2006 *n* = 27; 2007 *n* = 28; 2008 *n* = 1) completed the self-report questionnaire. Mean age as of the 25th of September 2017 was 11.5 ± 1.1 (range 9.3–13.7 years) and there was no statistical difference across the mean age of the five clubs (11.4 *n* = 38; 12.0 *n* = 4; 12.3 *n* = 13; 11.2 *n* = 19; 11.3 *n* = 22). The anthropometric characteristics of the racers during the pre-season testing are described in Table 1. The biological maturity status was not accounted for, however just 17.3% of the females (9/52) self-reported a regular menstrual cycle with 10 or more monthly periods per year and a mean onset of 12.0 ± 0.8 years. This age of menarche follows within the estimated mean range of 12–13 years in Canada (Al-Sahab et al., 2010).

**Table 1:** Anthropometric characteristics separated by sex (*N* = 96).

	**(Mean, 95% CI)**	
**Characteristic**	**F *n* = 52**	**M *n* = 44**
Age (yr.)	11.7 (10.5, 12.9)	11.9 (10.5, 12.9)
Height (cm)	151.5 (138.4, 160.5)	150.7 (137.1, 164.4)
Weight (kg)	41.7 (30.3, 49.6)	39.3 (27.4, 51.1)
WC (cm)	66.1 (58.8, 72.0)	65.3 (59.5, 74.1)
BMI (kg/m^2^)	18.1 (15.1, 20.3)	17.1 (14.6, 19.6)
PredictVO_2max_ (ml/kg/min^−1^)	49.2 (45.8, 52.5)	50.2 (45.0, 55.4)

*yr., year; cm, centimeters; kg, kilograms; kg/m^2^, kilograms per meters squared*;

*VO_2_ max, maximal oxygen uptake; ml/kg/min^−1^, milliliters per kilogram per minute; 99% CI, 99% confidence interval*.

Six levels of parental educational attainment were included in the questionnaire (junior high or less, high school, technical, undergrad, graduate or post graduate degree). The majority of the racers came from families where both parents (parent 1; *n* = 93; parent 2, *n* = 86) had a level 4 educational attainment (36.5%, *n* = 35; 27.1%, *n* = 26) or level 5 and 6 (above 39.7%, *n* = 37; 46.9%, *n* = 45) respectively.

### Physical and Activity Participation

The questionnaire was designed to capture hours of physical activity participation and categories of recreational activity. The 38 recreational activities were listed alphabetically in the questionnaire and included: Aerobics, Alpine skiing, Badminton, Baseball, Basketball, Boxing (incl. kick), Cross-country skiing, Cycling (road or mtn.), Dance, Dirt biking, Diving, Field hockey, Figure skating, Floor hockey, Football, Golf, Gymnastics, Hiking/Scrambling, Hockey, Horse riding, Lacrosse, Martial arts, Rock climbing, Rollerblading, Rugby, Running, Skate/long boarding, Snowboarding, Soccer, Squash, Speed skating, Swimming, Tennis, Track and field, Volleyball, Water polo, Weight training, Wrestling, with an additional open ended category of “Other.” Other sports recorded by the parents included: Climbing, Dryland Training, Kayaking, Pickleball/Parkour, Summer Camp, Water Skiing, Yoga, and not specified.

#### Past 6 Weeks of Sport and PA Participation Hours Outside of PE Class

In the 6 weeks prior to the start of pre-season training (4 weeks of August and first 2 weeks of September), 95% of the athletes (91/96) participated in sport or recreational activity outside of their PE class. Three males and 1 female reported that they did not, and one was blank. The majority (63.5%, 61/96) participated in 2–5 activities with a mean of 4.2 ± 2.4 (range 1–15) during this period. The most popular activities, regardless of sex, were cycling-road/mtn (77%), swimming (53%) and hiking/scrambling (45%) (see Table 2).

**Table 2:** Athlete participation in sport and activity during the past 12 months.

**Activity over past 12 months**	***N* = 96**	**%**
Alpine Ski Racing	96	100
Cycling (road/mtn)	55	57.3
Swimming	48	50.0
Hiking/Scrambling	35	36.4
Running	31	32.3
Soccer	24	25.0
Golf, Horseback riding, Rock climbing	16	16.7
Basketball,	12	12.5
Volleyball, Weight training	10	10.4
Gymnastics, Roller Blading	8	8.3
Badminton, Baseball, Dance, Skateboard, Track and Field	7	7.3
Marital arts	6	6.3
Floor hockey, Tennis	5	5.2
Aerobics, Diving, Figure Skating, Rugby	4	4.2
Cross-country skiing, Dirt Biking, Lacrosse, Squash, Wrestling,	2	2.0
Football and Other	1	1.0
Boxing (incl. kick), ice hockey, field hockey, snowboarding, speed skating, water polo	0	0.0

A range of hours per activity per week from 30 min to 56 h was reported. Only a handful of questionnaires had the level of intensity per activity session per week completed and when recorded the ranges included 1–10 mild, 2–7 moderate, and 1-vigorous.

Athletes who attended dedicated camps during the month of August recorded large volumes of daily activity during a week. These camps included: 1 week on site at the YMCA Camp Chief Hector (56 h), swimming (12–21 h), cycling (15–20 h), gymnastics (16 h), alpine skiing (13–15 h), volleyball (15 h), and golf (15 h). Not all activities from the list of 38 were participated in. This may be related to factors such as a lack of summer time offerings, proximity to location and interest in these activities (boxing including kick, ice hockey, snowboarding, speed skating and water polo).

#### Past 12 Months of Sport and PA Participation Hours Outside of PE Classes

In the 12 months prior to the start of the 2017 preseason training, 96% of the racers (*n* = 92/96) participated in sport or recreational activity outside of their PE classes (see Table 2). One male reported they did not, and three females left the section blank. The mean number of sports participated in with the inclusion of alpine skiing during the past 12 months was 5 (4.9 ± 2.8) and the number per athlete ranged from 1 to 14.

The cumulative total hours over the past 12 months were calculated from the hours per week multiplied by the number of weeks the athlete participated in the activity. There was no difference in PA participation between the male and female mean total hours reported (M 44, 389 ± 239; F 52, 398 ± 241), but the range was wider in the females (M 62–869; F 27–1,015 h) respectively, over the past 12 months.

#### Alpine Skiing Sport Participation Hours

The mean total hours reported over the past 12 months for alpine skiing in 91 athletes was 264.21 ± 170.2 (range 12–725). There were 12 athletes who reported over 480 h and 3 females had over 650 h. Both females and males on average completed 20 weeks of ski training (20.7 ± 7.69) with a range of 1–36 weeks, however the number of hours per week varied depending on the program category enrolled in. Racers select from 1 to 3 days per week of ski training with different combinations such as 3 full days which included both weekend days, or 2 full weekend days and a few hours one evening, or both weekend days, or only 1 day per weekend. The mean number of weekly hours was 13.43 ± 4.92 (range of 3–25), with females reporting slightly more (14.21 ± 4.73; range of 5–36) then the males (13.32 ± 5.36; range 3–24) hours. Some athletes participated in over 16 h per week of ski training.

### Injuries Were Reported by Activity Over a Lifetime and Over the Past 12 Months

#### Concussion

The number of concussions reported over a lifetime was 13 (13.5%; 3/96) and in the past 12 months was 4 (4.3%; 4/96). Males reported a higher prevalence of concussions (9/13) when compared to females (4/13). Alpine skiing had the highest prevalence of concussions (46.1%; 6/13) followed by mountain biking (15.4%; 2/13) over a lifetime. No memory loss or dizziness was reported however, those that did lose consciousness did so within the range of 3–10 s. On average, the number of days before return to sport was 14 (13.7 ± 6.5) with a range of 7–28 days. The majority of concussions were diagnosed by a physician (69.2%; 9/13) and two females (15.3%) reported they suffered from headaches post-concussion (see Table 3). Only one athlete reported having two concussion (F) over a lifetime, both related to playing tag during dryland training (2014).

**Table 3:** Reported concussions over a lifetime by sex separated by year, activity, return to sport and diagnosis (*n* = 13).

**Sex**	**Year**	**Activity**	**Unconscious seconds**	**RTS days**	**Physician DX**	**Headaches**
M	2017	Mountain biking		7	n	
M	2017	Mountain Biking		14	y	
M	2017	Alpine skiing		7	y	
M	2016	Scooter	5	14	y	
M	2016	Alpine Skiing	Not reported	14	n	
M	2016	Alpine Skiing	10	24.5	y	
M	2014	Alpine Skiing	3	7	n	
M	2014	Hockey		14	y	
M	2013	Not reported		14	y	
F	2017	Alpine Skiing		28	y	y
F	2014	Tag		14	y	y
F	2014	Tag		7	y	
F	NR	Alpine Skiing		14	n	

*RTS, Return to Sport; DX, Diagnosed; NR, Not reported; No memory loss or dizziness reported*.

#### Non-concussive Injuries Reported by Activity

The number of non-concussive injuries reported over a lifetime was 21.8% (21/96) and in the past 12 months was 16.6% (16/96) (Tables 4, 5). The return to sport range was 1–365 and this was influenced by two male athletes who required substantially more days to recovery from fractures in their lower leg (112, 365 days, respectively). As well, three injuries (back sprain, dental surgery and painful ankle) were reported as continuing to be problematic after resuming activities.

**Table 4:** Female non-concussive injury by year separated by body part and type and activity (*n* = 13).

**Date**	**Body part**	**Injury type**	**Activity**	**Treatment description**	**RTS days**
2017-01-15	Wrist	Sprain	Alpine Skiing	Physio	21
2017-02-01	Ankle	Sprain	Alpine Skiing	Rest, physio	4
2017-02-01	Knee	Strain	Cross-country skiing	Physio, ice, pain meds	7
2017-02-10	Knee	Sprain	Alpine Skiing	NR	60
2017-03-01	Ankle	Sprain	Gym Class	Physio, heat/ice	42
2017-03-03	Knee	Tweak [sprain]	Alpine Skiing	Physio	14
2017-03-10	Knee	Sprain	Alpine Skiing	Ice, pain meds	N/R
2017-05-01	Ankle	Sprain	Climbing	Ice	7
2017-07-01	Big toe	Fracture	Walking	Rest	20
2017-07-04	Wrist	Fracture	Walking-fell	Cast, brace	14
2017-07-27	Mouth	Teeth	Baseball	Surgery/Stitches, ice	15
2016-11-16	Knee	MCL Sprain	Alpine Skiing	Physio	42
2016-10-06	Wrist	Fracture	Bouncy Castle	Cast	12

*RTS, Return to Sport; NR, Not reported; Physio, Physiotherapy*.

**Table 5:** Males non-concussive injury by year separated by body part, type and activity (*n* = 8).

**Date**	**Body part**	**Injury type**	**Activity**	**Treatment description**	**RTS days**
2017-01-01	Neck	Sprain	Alpine Skiing	Physio	7
2017-04-07	Tibia	Fracture	Alpine Skiing	Cast, Physio	112
2017-08-15	Leg	Fracture	Alpine Skiing	Physio	365
2017-08-15	Back	Sprain	Trampoline	Physio	1
2017-08-25	Foot	Pain	Jumping off swing	NR	NR
2016-12-30	Shoulder	Sprain/Bruise	Alpine Skiing	Ice	2
2016-12-27	Knee	Strain	Alpine Skiing	Physio, Ice, pain meds	48
2016-03-06	Shin	Deep cut	Alpine Skiing	Stitches	21

*RTS, Return to Sport; NR, Not reported; Physio, Physiotherapy*.

Twelve non-concussive injuries over a lifetime (12.5%; F = 7, M = 6) were directly related to alpine skiing and the majority were related to lower leg injuries (F 9.6%, 5/52; M 9.1% 4/44 respectively). Females reported 11 (21.1%; 11/52) non-concussive injuries in the past 12 months and five were from alpine skiing compared to males who reported five (11.3%; 5/44) in the past 12 months with three related to alpine skiing. Only one athlete reported having two injuries (F), both related to walking in the same year (2017).

#### Physician Diagnosis of Previous Fracture, Arthritis, or Muscle Bone Condition

In addition to the above injuries, 15 fractures were reported with no information related to the activity: 10 in females (2015, growth plate in thumb and fibula; 2013 clavicle and left ankle, 2011 elbow and wrist; no date ankle and wrist) and 5 in males (2017 open growth plates, 2015 knee, 2014 arm and wrist). Therefore, the total number of injuries inclusive of the above (previous fracture 15, concussion 13 and non-concussive injuries 21) reported over a lifetime may be as high a 51.0%, (49/96).

### Medical History Related Medication and Supplements Use

The majority of the athletes (85.4%, 82/96) did not take medication on a regular basis and those that did (14.5%, 14/96) reported a medical and or health care professional diagnosis that included cognitive disorders, depression, and reactive airways and asthma (see Table 6). Male athletes (18.1%; 8/44) had a greater number of reported attention (ADHD, ADD, or combined; Dyslexia spectrum, Dyslexia and Dysgraphia) or learning issues (coded or anxiety, slow working memory or processing) when compared to females (11.5%; 6/52). The overall frequency of a diagnosed respiratory condition was 12.5% (12/96) with males (13.6%, 6/44) reporting slightly more cases than females (11.5%, 6/52). Only one athlete was diagnosed in 2017, the other cases were reported as occurring between 2007 and 2014 and were no longer considered active conditions.

**Table 6:** Medical History Diagnosis (*n* = 20) and medication use (*n* = 14) over a lifetime.

**Diagnosis**	**Medications reported**
ADHD (4), ADD (1)	Colchicine (1), Concerta (3), Vyvanse (1)
Learning Disorder (1)	Amitriptyline (1)
ADHD (1), Dyslexia spectrum (1), Dyslexia and Dysgraphia (1), Learning disability with dysgraphia (1)	NR
Depression (2)	Amitriptyline (1), Vyvanse (1), NR (1)
Diabetes (1)	Insulin
Asthma (4), Allergy (1)	Ventolin (2), Alvesco (1), NR (2)
NR (2)	Advil (1)

*ADHD, Attention deficit hyperactivity disorder; NR, not reported; ADD, Attention deficit disorder*.

In total, 53.1 % (51/96) of the athletes reported taking a supplement, of which most were vitamins and minerals (see Table 7). More females (59%; 31/52) took supplements when compared to the males (45.4%; 20/44).

**Table 7:** Reported use of supplements separated by sex.

**Supplement**	**F** **=** **52**		**M** **=44**	
	***n***	**%**	***n***	**%**
Vitamin D	11	21.1	7	15.9
Multivitamin	9	17.3	5	11.3
Omega 3	3	5.7	2	4.4
Probiotics	1	1.9	3	6.8
Magnesium			2	4.5
Vitamin C	4	7.6		
Vitamin A and B	1	1.9		
Calcium	1	1.9		
Melatonin	1	1.9		
Iron Feramax			1	2.2

### Health Care Utilization of Practitioner and Services

In the past 12 months 25% of the males (11/44) went to emergency for a variety of reasons including but not limited to a bruised shoulder, a fall, kidney issue, lip surgery, stomach pain, and tibia fracture. Females (25%, 13/52) went to emergency for similar reasons (asthma, concussion, face injury, headache (2), knee injury (3), pneumonia, stomach pain, and wrist fracture), however no females were admitted for an overnight stay. Over their lifetime males (*n* = 5) had more surgery including repair of undescended testicle and circumcision and ruptured appendicitis that included a 5-night stay with a 42 day return to sport. Males were also admitted for at least one night in the hospital that included: ear, nose, and throat referral; concussion and asthma-2-night stay.

A large portion of the athletes (69.2% F; 65.1% M) had contact with their family physician in the past year. Females reported a slightly greater number (*n* = 102) of allied health care, sport medicine visits and x-rays appointments when compared to males (*n* = 65) (see Table 8).

**Table 8:** Practitioner utilization over the past 12 months (excluding hospitals visits) separated by sex.

**Practitioners and services**	**F** **=** **52**		**M** **=** **44**	
	***n***	**%**	***n***	**%**
Physician—emergency room doctor	14	26.9	14	31.8
Physician—general practitioner/ family	36	69.2	28	63.6
Physician—sport medicine doctor	4	7.7	1	2.3
Surgeon	0	0.0	4	9.0
Physiotherapist	10	19.2	3	6.8
Chiropractor	8	15.4	3	6.8
Massage therapist	9	17.3	4	9.0
Athletic therapist	1	1.9	1	2.3
Magnetic resonance imaging (MRI)	2	3.8	0	0.0
Computer tomography (CT scan)	3	5.8	0	0.0
Radiographs (x-rays)	11	21.2	7	15.9
Other (please specify)	4			
**Total no of visits**	**102**		**65**	

## Discussion

We report on 96 athletes who represent 26.4% of the 363 ski racers registered across all clubs in the AASA age groups of U12 and U14 in 2017 (http://albertaalpine.ca/wp-content/uploads/2017/05/AASA-Membership-Statistics-2017.pdf). When compared to a group of provincial level Austrian ski racers (mean age M 12.3 ± 1.2, F 12.4 ± 1.3 yrs.; body weight (kg) M 44.1 ± 9.2, F 46.8 ± 10.8; height (cm) M 153.6 ± 9.4, F 155.4 ± 9.6; and BMI (kg/m^2^) M 18.5 ± 2.2, F 19.1 ± 2.6), our racers (see Table 1), were younger, lighter and not as tall (Müller et al., 2016). Younger age and a club style of training vs. attendance at a full-time ski school academy likely contributed to these differences in body composition.

Ski racers in Western Canada are primarily supported by their parents and coaches working in collaboration to ensure they have the best opportunity to achieve their potential. The cost burden of competing is paid entirely by parents through the club model typical of Canadian ski racing. Clubs are incorporated under the Societies Act which permits some retained earnings but otherwise requires dollars to be spent toward declared objectives. These objectives include payment of coaches through salaries, gym rental, on hill training such as lane space, equipment etc., and all of these costs are passed on to parents and subsequently recovered from their registration fees. Therefore, have high levels of education because of the association with better economic outcomes is both common and a necessity for parents of racers. This higher level of education and higher household income is not just associated with ski racing (PBM, 2016), but with all sport participation in Canada (Statistics Canada, 2013a,b).

### Physical Activity Volume

The primary aim of this present study was to describe the participation of all sport and physical activity outside of physical education classes over three timelines: just prior to the start of pre-season training, over previous 12 months and lifetime. We know that Canadian children (ages 5–12 years) spend a considerable amount of time participating in sports and recreation activities, particularly in the summer months (CFLRI, 2009). These athletes were no different as observed by the large range of activities which included several summer camps within the 6 week period prior to start of the September school year. In terms of commonly participated activities in adolescents, the Canadian Sport Participation Report, lists soccer followed by swimming and ice hockey (2010). However, our athletes, regardless of whether they were female or male, participated in cycling-road/mtn (77%), swimming (53%) and hiking/scrambling (45%). These three activities are associated with warmer weather in Alberta, which is more often in the late summer months of August and September. Many of these athletes also live within close proximity to the mountains, which likely influence their participation in hiking and mountain biking because of the associated physical and social-cultural environment (Bolívar et al., 2010).

The daily recommended amount of physical activity (365 h/year) is not given as an accumulated index of exposure or total volume over a 12 month period, which is a standard in injury surveillance research (Nielsen et al., 2019). However, these athletes self-reported just under 400 h of activity (range females 27–1,015; males 62–869 h), and therefore easily meet both the World Health Organization (2010) and Canadian recommended 60 min of daily PA for their age group (2018).

The athlete's total participation hours included dedicated time on snow (November-May), which from a skill acquisition is important during the adolescent years based on Alpine Canada's program LTAD (2019). The Training and Competition Focus Matrix states that U10 should have 10–15 days on snow with 50–65% free ski volume training and U14 should have 15–30 days on snow with 40–50% of their training time dedicated to technical and tactical skiing skills. Our racers easily met these criteria with an average of 20 days on snow and much of their training involved off-piste skiing because of the positive snow and terrain conditions in the Rocky Mountains.

To be a strong and fast skier at the elite level requires participation in a variety of training forms outside of on snow training (Gilgien et al., 2018). The current literature related to young athletes also focuses on avoiding early specialization (Post et al., 2017). Somewhat surprisingly, 15.5% (15/96) of the athletes reported over 480 h of skiing over the previous year. Three of these athletes (F) reported 650 plus hours of dedicated alpine skiing, and this could be viewed as an excessive amount for 10–14 year old's, even if it is at a low intensity (Faigenbaum, 2019). The highest total volume of PA at 1,015 h was reported by a U14 female athlete which included the second most total hours of alpine skiing (720 h). This athlete participated in a variety of activities that included badminton (5 h), figure skating (30 h), golf (192 h), tennis (15 h), and weight training (32 h). Like others she also did cycling (20 h) and running (1 h). She sustained a lower body injury (MCL strain) in the following year (2018) that required 4 weeks of physio before she returned to activity. Launay (2015) states that overextending physically, can result in overuse injuries to the musculoskeletal system and perhaps this large volume in weekly sports and activities in combination with ski training and competition contributed to her injury. This outcome is supported by Räisänen et al. (2016) in a study that showed higher PA participation frequency and intensity increased the risk of injury and that injury prevalence was typically highest in sports club activities (2016).

### Fundamental Movement Skills

Developing skiers require a large FMS repertoire which includes training of coordination /motor control, balance and quickness. This type of “training smarter” involves off snow imitation of skiing associated with a variety of activities such as cycling, running (on uneven terrain) and soccer (football) (Raschner et al., 2004; Gabbett, 2016). All ski clubs in this study encouraged athletes to participant in activities outside of skiing and the mean number of those participated in by our athletes was four. Clubs also incorporated strength and balance activities into their dryland and preseason training programs beyond just running and games of soccer. Recent research states that cycling, one of the favorite activities among our athletes not only contributes to balance but is also a cognitively-engaging exercise and has a stronger effect on executive function when compared to other aerobic exercise (Best, 2010; Leyland et al., 2019). Therefore, the athletes in the ski club system were exposed to a variety of activities outside of on-snow training, helping to develop their FMS proficiency, which is advocated for in the Canadian Alpine LTAD model.

### Injuries

The secondary aim of this study was to provide insight into the injuries reported and health indicators in adolescent youth ski racers over the past 12 months or a lifetime. In Canada, two out of three (66%) injuries among adolescents reportedly are linked to sport and this age group has a higher percent of fractures (21%), lower limb (33%) hand and wrist (22%) injuries when compared to older adults in Canada (Billette and Janz, 2015). Most growth plate fractures happen from falling and twisting and are common in fast moving sports such as skiing and biking (Malina et al., 2004). Therefore, it is not surprising based on our athletes' activities that there were several reported cases of these injuries (see Tables 4, 5) with the knee and ankle as the most common locations (Malina et al., 2004).

It is interesting to note that ice hockey, rugby and ringette are sports with the highest proportion of brain injuries among children aged 5–19 years (CHIRPP, 2018). Due to a growing concern around concussion management in youth alpine skiing, the AASA implemented an updated concussion policy effective in the 2016–17 seasons. This policy stated that if a coach suspected a concussion then the athlete should be immediately removed from the hill and their pass suspended until physician clearance was received by the organization. It also mandated coach education and required parents to sign that they acknowledge the policy. This may have resulted in greater awareness and as such the number of concussions (4%) reported by athletes was considerably less than other non-concussive injuries (16.6%) reported in 2017.

#### Diagnosis of Attention Deficit-Hyperactivity Disorder (ADHD) and Learning Issues

Sports in general provide a positive experience for adolescents with ADHD with evidence showing a statistically significant decrease in markers of anxiety and depression with higher levels of sports participation (Kiluk et al., 2009; Perrin and Jotwani, 2014). The male athletes had a greater number of reported attention or learning issues when compared to females in our study. The prevalence of ADHD in student and elite athletes is between 7 and 8% (Han et al., 2019) with symptoms often surfacing just prior to the age of 12 (Thomas et al., 2015). Given that there is a positive experience with sport participation the prevalence in youth athletes maybe greater than in the general population (Poysophon and Rao, 2018). To the best of our knowledge this is the first reporting of the prevalence ADHD in young alpine skiers.

#### Reactive Airway and Asthma

Airway disease has been reported in the literature as the most frequently encountered chronic respiratory condition in athletes (Hull et al., 2012) however, there are only a few reported studies related to alpine skiing. The prevalence of exercise-induced asthma (EIA) or exercise-induced bronchoconstriction (EIB) occurs in about 15% of cross-country skiers in comparison with a <4%, in alpine skiing and ski jumping despite training in similar weather conditions (Karjalainen et al., 2000). Asthma is the most common chronic disease affecting children in Alberta and EIA is a general concern during growth and development according to The Wellbeing Report of Canada's Young Children (2011). Our athletes reported a frequency (12.5%), more similar to cross-country skiers however Alberta is known for very dry air conditions. Therefore, it may not be unexpected to have a greater prevalence of EIA or reactive airway conditions in young alpine skiers given the amount of time they spend in the outdoors.

#### Supplements

Supplemental vitamin and mineral (VM) use are common among adult Canadians, and more prevalent among those with healthier lifestyles and of socio-economically advantaged backgrounds (Guo et al., 2009). According to Health Canada: (2015) supplement use in those aged 9–13 years is 36.8% and teenage females use more than males (32.9% vs. 26.5%). The province of Alberta has the highest rate of VM supplemental use among children and teenagers at 54.1% (Health Canada, 2015). Our study results demonstrate similar trends as the above with over the half the athletes (53.6%) taking supplements and female (59%) consuming more than the males 46.5%.

#### Utilization of Practitioners and or Services

Previous research shows that participating in organized sports is a major risk factor for hospitalization throughout adolescence (Mattila et al., 2009) and the Canadian sport and athletic statistics identify males as being hospitalized more often due to injury than females (National Ambulatory Care Reporting System 2017–2018, 2019). A similar a pattern of emergency room visits occurred with our athletes but with limited overnight stays and those that were hospitalized included only males (CIHI, 2019). The emergency room visits involved sport and activity related injuries as well as a variety of other health reasons. Given our athletes ages this is not unusual as the top pediatric visits, other than injuries, typically include poisonings, breathing problems, neurological, infections and gastrointestinal problems; all of which, with the exception of poisoning these athletes experienced.

The majority of the athletes (68%, *n* = 64) visited their family physicians in the past year. This was slightly greater than the Canadian frequency of 60% in the age group of 12–17 years old (Statistics Canada, 2018). Generally, provider contact in this age group is related to non-preventative care visits vs. preventive care visits (Nordin et al., 2010), and many of the athletes in this study saw other providers for a variety of treatment care (athletic therapist, chiropractor, massage therapist and physiotherapist).

## Strengths and Limitations

To the best of our knowledge, this is one of only a few studies (Müller et al., 2017c), investigating self-report PA, injury and illness health surveillance data in this age group of alpine skiers. However, information bias often accompanies self-report data and previous literature has shown that a short recall period is preferable to a long one, particularly when asking participants about routine or frequent events (Althubaiti, 2016). Our questionnaire had three time periods which may have resulted in overlap of the reporting and given this we should have paid more attention educating parents on how to complete the forms. This may have reduced some of the missing data and improved the quality of data particularly related to intensity of activity, which was poorly filled in.

We recognize that the alpine skiing community is interested in understanding the impact and timing of training load which has been identified as a valuable modifiable risk factor for injury (Gabbett, 2016). Our volume calculation of PA was based on the number of hours spent each week in ski training and other recreational physical activities outside of PE classes, and is at best a crude approach, although practical. However, if volume could be combined with a perceived exertion rating, determination of training load could occur (Wallace et al., 2017). This combination of volume and intensity of activity (total load) should be considered since many 10–14 year old athletes are entering their growth period which is associated with an increased risk of overuse injuries (Bahr, 2014; Jayanthi et al., 2015). Feasibility is also important and therefore future research should consider wearable devices to measure PA volume objectively with accelerometers and or in combination with a Global Positioning System so to measure how individual training loads change over time (Drew and Finch, 2016).

Lastly, the use of common measures for the assessment of modifiable risk factors and health outcomes is necessary for future comparison. In this cross-sectional study the data collection occurred through each ski club, which was very labor intensive from the researchers and the club personnel perspectives. Similar to previously published studies in winter sports (Niedermeier et al., 2019), we acknowledge the limitations connected to a cross-sectional study based on self-reports (e.g., impossible to assess causal relationships, non-truthfully answered questions, or a potential recall bias). The implementation of a centralized system through the ASSA or Alpine Canada Association with an online version of the baseline medical questionnaire would streamline the process.

## Conclusion

In conclusion, adolescent alpine ski racers in Western Canada were exposed to many hours of physical activity and participated in several different sports outside of ski training and physical education classes; all of which contribute to their fundamental movement skills. The accumulated volume of physical activity from ski training over the previous 12 months and all other physical activities was higher than the Canadian recommended guidelines of 60 min of daily PA for their age group (2018). This study did not report on intensity of physical activity and therefore future research should consider monitoring the combination of volume and intensity to identify whether this age group of ski racers could be at risk of overextending themselves physically.

The outcomes from the baseline medical questionnaire highlighted a typical pattern of injuries, common health issues and practitioner visits related to this age group when compared to the population at large. However, athletes that suffered an injury did lose participation hours particularly when related to lower leg injuries, and therefore strategies to prevent these injuries is an important consideration (Öztürk and Kılıç, 2013). Future research directions should consider an easily accessible on-line health and injury surveillance questionnaire in combination with wearable technology so as to better quantify training load across all sport and physical activities in 10–14 year-old Canadian alpine ski club racers.

## Data Availability Statement

The datasets generated for this study are available on request to the corresponding author.

## Ethics Statement

The studies involving human participants were reviewed and approved by The Conjoint Health Research Ethics Board (CHREB), University of Calgary reviewed and approved the following research protocol: Ethics ID: REB16-1818. Written informed consent to participate in this study was provided by the participants' legal guardian/next of kin.

## Author Contributions

PD-B designed the study, collected and analyzed the data. CE contributed to study proposal development, contributed to study design and critically reviewed the edited manuscript before submission.

## Conflict of Interest

The authors declare that the research was conducted in the absence of any commercial or financial relationships that could be construed as a potential conflict of interest.
